# Recent Applications of Three Dimensional Printing in Cardiovascular Medicine

**DOI:** 10.3390/cells9030742

**Published:** 2020-03-17

**Authors:** Chiara Gardin, Letizia Ferroni, Christian Latremouille, Juan Carlos Chachques, Dinko Mitrečić, Barbara Zavan

**Affiliations:** 1 Maria Cecilia Hospital, GVM Care & Research, 48033 Cotignola (RA), Italy; chiara.gardin@unife.it (C.G.); letizia.ferroni@unife.it (L.F.); 2 Department of Medical Sciences, University of Ferrara, via Fossato di Mortara 70, 44121 Ferrara, Italy; 3 Department of Cardiac Surgery Pompidou Hospital, Laboratory of Biosurgical Research, Carpentier Foundation, University Paris Descartes, 75105 Paris, France; Christian.Latremouillec@gmail.com (C.L.); j.chachques-ext@aphp.fr (J.C.C.); 4 Laboratory for Stem Cells, Croatian Institute for Brain Research, School of Medicine University of Zagreb, Šalata 12, 10 000 Zagreb, Croatia; dinko.mitrecic@mef.hr

**Keywords:** 3D printing, 3D model, bioprinting, cardiovascular medicine, heart, myocardium, heart valves

## Abstract

Three dimensional (3D) printing, which consists in the conversion of digital images into a 3D physical model, is a promising and versatile field that, over the last decade, has experienced a rapid development in medicine. Cardiovascular medicine, in particular, is one of the fastest growing area for medical 3D printing. In this review, we firstly describe the major steps and the most common technologies used in the 3D printing process, then we present current applications of 3D printing with relevance to the cardiovascular field. The technology is more frequently used for the creation of anatomical 3D models useful for teaching, training, and procedural planning of complex surgical cases, as well as for facilitating communication with patients and their families. However, the most attractive and novel application of 3D printing in the last years is bioprinting, which holds the great potential to solve the ever-increasing crisis of organ shortage. In this review, we then present some of the 3D bioprinting strategies used for fabricating fully functional cardiovascular tissues, including myocardium, heart tissue patches, and heart valves. The implications of 3D bioprinting in drug discovery, development, and delivery systems are also briefly discussed, in terms of in vitro cardiovascular drug toxicity. Finally, we describe some applications of 3D printing in the development and testing of cardiovascular medical devices, and the current regulatory frameworks that apply to manufacturing and commercialization of 3D printed products.

## 1. Introduction

Three dimensional (3D) printing is a technique used to transform digital images in a physical 3D model by fusing or depositing material layers. The materials deposited can be powders, plastics, ceramics, metals, liquids, or even living cells, making the process extremely versatile [1,2]. The first technology for 3D printing, called stereolithography, was introduced in 1986 by Charles Hull [3]. From its invention, 3D printing has been largely developed, mostly in the last decades, and nowadays several techniques are available, with applications spanning from the industrial to the medical field [4].

In medicine, 3D printing is utilized for several purposes such as teaching, surgical planning, development of novel and/or personalized implantable devices, and also for creating scaffolds for tissue engineering and artificial functional tissue regeneration [5]. Since its first introduction, the application of 3D printing has greatly expanded mainly in the maxillofacial and orthopedic sectors [6]. With regard to the cardiovascular field, one of the most popular clinical uses of 3D printing is related to the possibility to create 3D printed heart models. These personalized models are proven to be particularly useful in pre-operative planning and pre-surgical simulation of complex cardiac interventions, intra-operative orientation for improving clinical decision-making, medical education and training, and communication in medical practice [7]. In this review, we firstly introduce the 3D printing process and technologies with relevance to cardiovascular medicine. Then, we present some cases of patient-specific 3D printing applications in cardiovascular pre-operative training and pre-surgical planning. Since 3D bioprinting currently represents the most attractive application of 3D printing in the healthcare sector, we then introduce methods for 3D bioprinting and the most commonly used bioinks. This review subsequently covers the applications of 3D bioprinting in the cardiovascular field through categories that include myocardium, heart valves, and cardiac patches for drug screening. In the last section, we describe current regulatory frameworks that USA and EU apply to 3D printed products. Finally, we summarize the major limitations of 3D printing and bioprinting, and the future directions that will enable the translation of these technologies to personalized therapeutic and pharmaceutical applications.

## 2. Process and Technologies of Cardiovascular 3D Printing

**Figure 1:**
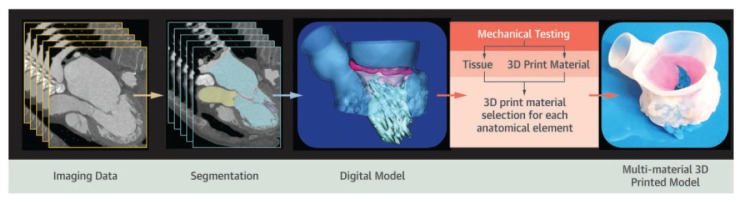
Cardiovascular 3D printing workflow includes acquisition of imaging data, segmentation, imaging modeling, and actual 3D printing. Reprinted with permission from Vukicevic et al. [12]. Copyright © 2020 American College of Cardiology Foundation.

Generating a 3D model is a complex process comprising the sequential stages of diagnostic images acquisition, digital modeling, and 3D printing (Figure 1) [8]. Close collaboration between physicians, imagers, and engineers is therefore fundamental to obtain a functional and accurate 3D printed model.

The first step in the 3D printing process is the acquisition of accurate volumetric images formed by contiguous multiple slices that provide a dataset. Medical images suitable for 3D printing must have high contrast between adjacent structures, low noise, and high spatial resolution [9]. The methods usually employed to acquire cardiovascular imaging data are computed tomography (CT) and magnetic resonance imaging (MRI), but in some cases also 3D transthoracic echocardiography (TTE) or 3D transesophageal echocardiography (TEE) are utilized [10]. Since the quality of the imaging sourcing data is fundamental to obtain precise 3D models, it is essential to evaluate the advantages and limitations of each imaging modality prior to acquiring patient images for 3D modeling. CT represents the preferred imaging technique for 3D printing, because it can provide sub-millimetrical resolution of tissues. In the cardiovascular field, CT is an advantageous option for modeling both intracardiac (atria and ventricles) and extracardiac (great vessels) structures [11]. In addition, CT is able to clearly identify bone and pathologic calcium deposition, and to image patients with pacemakers, artificial valves and metal implants that are not compatible with MRI scanning [12]. However, the major limitation of CT is the exposure to radiation caused by the emission of X-rays, which has been correlated with increased risk of cancer [13]. On the other hand, MRI allows the acquisition of high-resolution images without the employment of ionizing radiation. This imaging modality is mostly employed for visualizing soft tissues; for example, it has been used for the creation of heart chambers and vasculature 3D models, and for the reconstruction of intracardiac tumors [14]. However, a limitation of MRI in comparison to CT includes a lower spatial resolution that limits its use for the evaluation of coronary arteries or the small morphological features within heart valve complexes [12]. 3D echocardiography is a widely available, relatively low-cost ultrasound imaging technique which, similarly to MRI, lacks ionizing radiation. 3D echocardiography is a valuable tool to 3D model ventricular chambers, cardiac valves, and the interatrial septum; however, it is subjected to artifacts and it is not suitable for visualizing extracardiac structures, such as the aorta and pulmonary arteries [15].

The second step in the 3D printing workflow is segmentation, namely, the delineation of appropriate regions of interest for creating patient-specific and highly accurate models of organs and tissues. In cardiovascular 3D printing, this process aims at discriminating the cardiovascular structures of interest, excluding irrelevant noncardiac structures, such as bone and lung [9]. Segmentation is a multi-step process. Prior to segmentation, the acquired diagnostic imaging dataset is exported into a digital imaging and communication in medicine (DICOM) format, which can be universally used in digital modeling softwares without the need for file type conversion [16]. Several medical image segmentation softwares are available; some of these are open-source and freely accessible, while others are commercial, licensed products. From the DICOM dataset, the target anatomic geometry is identified and segmented based on properties such as contrast or brightness [17]. As a result, segmentation masks are created such that pixels with the same intensity range are grouped and converted into 3D digital models using rendering techniques. These segmented digital models are then exported out in the standard tessellation language (STL) file type, which is the industry standard for 3D objects and 3D printing software [17]. Nevertheless, STL files do not contain color information and in order to print in multiple colors, a different file format will need to be used. For example, virtual reality modeling language (VRML) and additive manufacturing file format (AMF) provide multiple color and material options [18]. Once segmentation is completed, the final 3D digital model may be further modified within computer-aided design (CAD) software. The main post-processing adjustments of the 3D model are: Repairing errors and discontinuities that sometimes arise in the image segmentation and exporting processes, smoothing of the surface of the model due to scaling errors resulting from the resolution of the original medical image, and addition of other structures or elimination of unneeded parts from the segmented model [19].

The last step is the actual printing, and in this context it is important to identify the best type of technology and materials in relation to the final purpose, considering complexity, durability, and quality of the model to be obtained. Currently, several 3D printing technologies are available; the most frequently reported with potential applications in cardiovascular medicine are stereolithography (or vat polymerization), powder bed fusion (or selective laser sintering), material extrusion (or fused fiber filament/fused deposition modelling), and material jetting (or polyjet printing) [10,19]. Stereolithography and powder bed fusion technologies use a laser to fuse multiple layers of print material [17]. In particular, stereolithography uses ultraviolet laser light to harden the surface layer made of photopolymeric liquid resin, whereas powder bed fusion uses a laser to heat and fuse a bed of powdered printing material without requiring any support structure. In contrast, material extrusion and material jetting technologies use a nozzle or jet, respectively, to lay down a liquefied print material, which then solidifies before a new layer is built [17]. In detail, the extrusion technique creates 3D models by extruding thermoplastic materials or bioinks filaments layer by layer. Material jet printers, instead, produce 3D models by jetting thin layers of photopolymers that are instantly hardened by ultraviolet light, and allow the incorporation of multiple-materials and colors simultaneously. Once the print is completed, post-processing may also be required, which often includes cleaning to remove residual debris and support material and sanding if smooth surfaces are needed [14].

## 3. 3D Printing for Teaching and Surgical Training in Cardiovascular Medicine

**Figure 2:**
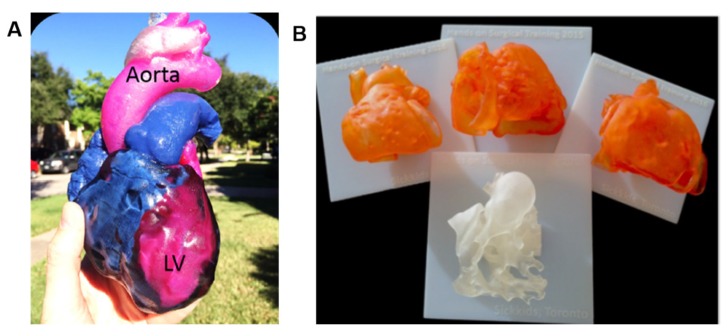
(**A**) Multi-material and multi-colored patient-specific 3D printed heart for educational purposes and communication with patients. LV, left ventricle. Reprinted with permission from Vukicevic et al. [12]. Copyright © 2020 American College of Cardiology Foundation. (**B**) Four models for surgical practice and training. Reprinted with permission from Yoo et al. [18]. Copyright © The Author(s) 2016.

In modern medical schools, the use of 3D models is emerging as a successful novel tool to support and complement the classic methods for anatomy teaching and demonstration [20,21]. The creation of 3D models starting from a clinical dataset of images allows the realization of teaching models of normal and abnormal anatomy. Traditionally, cardiac morphology teaching is carried out using samples collected during cadaveric dissection or heart transplantation in pathological patients [18]. Nonetheless, the scarcity of these specimens, associated with the fact that these do not represent all possible heart diseases, makes 3D models more promising educational resources [18]. Interestingly, 3D models can be fabricated with materials that mimic the real consistency of tissues and they can be scaled to any size. In addition, these 3D models can be printed with different colors, variable material hardness, and even layered texturing if needed to reproduce sophisticated or unusual cardiovascular pathology (Figure 2). Another aspect not to be underestimated is the possibility for several generations of students to handle, manipulate and experience such anatomical variations because 3D printed models are reusable and safe. Consequently, there is no need of special laboratories, instruments, or equipments, such as cadaveric specimens, whose availability is becoming increasingly difficult due to logistical, financial and ethical issues [21,22,23,24].

3D printed anatomical models are great resources also for surgical and pre-operative training, thanks to the patient-specific information which can be obtained from the model. In particular, 3D printed models are considered more helpful than virtual reality (VR) and 3D digital images when visualizing complex cases. Indeed, VR and 3D digital imaging do not always demonstrate spatial relationships efficiently and may not always provide an accurate representation of the anatomy [24,25]. The use of such 3D printed models allows a realistic simulation of the surgical procedure to be performed before the actual surgery, thus acting as a surgical guide. If, on one hand, the trainees can learn, practice and repeat surgical procedures until they feel confident, the experienced surgeons, on the other hand, can use 3D printed models for developing new procedures or improving their surgical skills for rare diseases. This is especially true for certain rare congenital heart diseases (CHD), where each patient has a different intracardiac and extracardiac anatomy, such as the chest wall and the relation of the heart to the chest wall. In all these cases, 3D printed models can lead to better pre-operative planning [26,27]. Other advantages of training using 3D models include a reduction in operation time and the possibility to predict intra-operative complications and plan management as required [21]. The use of 3D printed models can ease the communication not only within the clinical staff but also with patients and their family [28]. For example, the patient’s cardiovascular pathology and the intended or previously performed surgical procedure can be more easily understood when it is explained using 3D printed models [18].

Several cases of patient-specific 3D printing applications in cardiovascular pre-operative training have been reported. Recently, Bateman and coworkers at the University of Minnesota described three case studies in which heart 3D printed models resulted fundamental to evaluate the most appropriate surgical procedure [14]. The first case concerned a patient with dextrocardia that needed the placement of a left ventricular epicardial pacing. Because of the patient’s anomalous anatomy, a 3D model was requested to highlight the paths of the coronary arteries across the myocardium of the left ventricle (LV), finally permitting to practice a minimal invasive surgical approach. The second case was about a patient with a very complex congenital heart disease accompanied by a complex vascular anatomy that requested heart transplantation. The creation of a detailed 3D printed model allowed to determine the best donor and recipient preparation to plan for the implantation of a normal anatomic heart into an anomalous cardiac vascular anatomy. In the last case study, two conjoined sisters attached from chest to navel had to be separated. 3D printed models of the two hearts revealed a previously unseen myocardial connection and led the surgeons to change the surgical procedure. These three case studies demonstrate that the close collaboration between the University of Minnesota’s Academic Health Center and relevant departments of engineering has created an excellent environment for developing projects based on the clinical application of 3D printing in the cardiovascular field.

Patients with non-valvular atrial fibrillation (AF) undergoing left atrial appendage (LAA) occlusion can benefit from the advantages of 3D printing. LAA occlusion represents an alternative to anticoagulation for stroke prevention in patients with AF. In the retrospective and prospective analysis described by Fan and colleagues on 107 patients undergoing LAA occlusion, the creation of patient-specific 3D models from 3D TEE imaging data was associated with improved procedural safety and efficacy [29]. In particular, the use of such models offered outcome-relevant information anticipating the complexity of the surgical procedure, permitting to choose the best size device, thus improving intraprocedural performance and safety.

Motwani and colleagues reported a case where a 63-year-old man, several years after a cardiovascular intervention, developed a severe aortic regurgitation (AR) and needed to be re-operated for the closure of paravalvular leak (PVL) [30]. After evaluation with coronary angiography and echocardiography, the multi-disciplinary team decided to proceed with the transcatheter percutaneous closure of the PVL, which is a relatively novel procedure characterized by a small range of closure devices and limited sizing guidance. The creation of 3D models from CT images allowed to test different closure devices and to evaluate the best anatomical fit without interference with adjacent structures. After these bench tests, surgeons acquired high pre-procedural confidence with a particular closure PLD-W device. At 2 months from the intervention, the device remained well-seated with only trivial AR, and the patient returned to his active lifestyle without symptoms.

Over the last years, 3D printing has been demonstrated particularly helpful in the pre-surgical planning of minimally invasive procedures, such as mitral valve (MV) repair surgery [31,32,33]. Minimally invasive procedures are considered as very complicated, and reconstructive MV surgery is particularly challenging due to variation in valve pathologies. In addition, the possibility of surgeons to practice is limited and most of the pre-surgical training is conducted by assisting and performing real operations [34]. Various surgical repair techniques exist (e.g., annuloplasty, leaflet plasty, chordae plasty), which may address different parts of the valve [35]. Vukicevic and coworkers created patient-specific 3D MV models with different materials starting from TEE and CT imaging datasets. The authors stated that the material properties of such models facilitated functional evaluation of novel trans-catheter MV repair strategies, thus providing surgeons a realistic tool for surgical training [31]. More recently, Engelhardt and colleagues produced for the first time 3D printed MV silicone models containing the full MV apparatus of specific patients, that is the annulus, leaflets, chordae tendineae, and papillary muscles [33]. Then, twelve surgeons performed various surgical techniques, such as annuloplasty, neo-chordae implantation, and triangular leaflet resection, judging the realism of the valves very positively.

**Figure 3:**
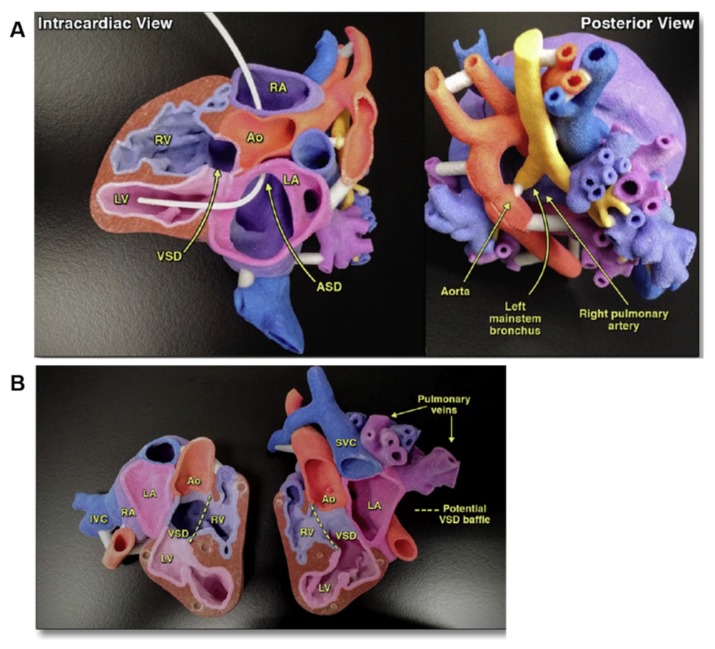
(**A**) 3D model printed to visualize the complex anatomy and aid surgical planning of a four-month-old patient with superoinferior ventricles, atrial septal defect (ASD), ventricular septal defect (VSD), left lung collapse, leftward shift of the heart, and compression of the left mainstem bronchus. (**B**) A 3D model obtained from cardiac MRI of an eight-month-old patient with situs inversus, dextrocardia, double-outlet right ventricle (DORV), and L-malposed great arteries was printed to guide next steps in surgical management. Reprinted with permission from Anwar et al. [39]. Copyright © 2020 Elsevier.

As mentioned above, 3D printed models result particularly helpful for a variety of complex CHD, including double-outlet right ventricle (DORV), atrial septal defect (ASD), and ventricular septal defect (VSD). Treatment of these conditions is generally surgical closure, although percutaneous approaches with closure devices are considered a safe alternative [10]. 3D printing has valuably aided in the spatial navigation of occluder devices during the operation and in optimizing patch sizing. In last years, a broad range of complex congenital heart anatomies have been reconstructed and 3D printed to enhance surgical planning (Figure 3) [36,37,38,39]. The Royal Brompton Hospital was the first to use images from cardiac MRI to replicate signs of scarring in 3D heart models for better understanding and improving treatments for CHD patients with arrhythmias [28]. In their models, scar tissues are printed with rigid, colored material for better visualization, allowing cardiologists to decrease invasive diagnostic cardiac catheterization procedures and to choose the best surgical procedures in relation to the patient conditions.

Despite the evidence that 3D printing could be very useful in teaching and clinical consultations, as well as in pre-operative planning and decision-making, additional studies are needed to determine whether 3D printed models are cost-effective and can reliably improve clinical outcomes before they become part of routine clinical practice.

## 4. 3D Bioprinting for Cardiovascular Applications

The increase of organ requirement for transplantation and the ever-decreasing availability of donor has driven research to try to create biological substitutes mimicking native tissues to restore or replace multi-functioning organs. Therapies based on tissue engineering and regenerative medicine represent a potential solution for the organ donor shortage [40]. The traditional tissue engineering strategy is based on the incorporation of stem cells into natural or synthetic scaffolds that, in the presence of appropriate growth factors, direct cell proliferation and differentiation into 3D functioning tissues [41]. This approach has generated some significant successes in the past decades both in research and clinical applications, including myocardial tissue, vessel, and heart valve [42,43,44,45]. Nevertheless, complex 3D organs, such as kidney, liver, lungs, and heart, require precise multi-cellular structures with vascular network integration.

The new frontier of tissue engineering is to exploit 3D printing for functional organ reproduction. For this purpose, a special 3D printing method, named bioprinting, has been developed. 3D bioprinting was first introduced by Klebe in 1988, who used the term ‘cytoscribing’ for describing the technique of the precise placement of cells on a substrate using a computer-controlled inkjet printer or graphics plotter [46]. Currently, bioprinting refers to the 3D printing of biocompatible materials, cells, and biomolecules into complex 3D functional living tissues [47]. The bioprinting fabrication process is highly controllable, allowing precise positioning of various biomaterials and living cells simultaneously according to the natural compartments of the target tissue or organ. When compared with traditional tissue engineering techniques, 3D bioprinting offers additional important advantages, such as high precise cell placement and high digital control of speed, resolution, cell concentration, drop volume, and diameter of printed cells [48,49].

**Figure 4:**
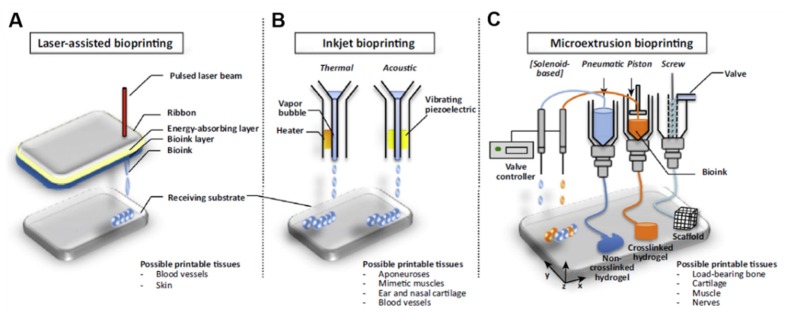
Schematic representation of 3D bioprinting technologies. (**A**) Laser-assisted bioprinting, (**B**) inkjet bioprinting, and (**C**) microextrusion bioprinting. Reprinted from Visscher et al. [50]. Copyright © 2020 Elsevier Ltd. All rights reserved.

Based on the working principles and depending on the type of tissue to be fabricated, methods for 3D bioprinting include laser-assisted bioprinting, inkjet bioprinting, microextrusion bioprinting, and integrated approaches (Figure 4) [50]. Inkjet-based bioprinting, the most common method for 3D printing of live cells, is a non-contact technique in which living cells are printed in the form of droplets through cartridges instead of seeding them on scaffolds [49]. The mechanism used to generate the picolitre droplets can be thermal, piezoelectric, laser-induced, or based on pneumatic pressure. Thermal inkjet bioprinting has been shown to be more biocompatible to the living system comparing to the other inkjet bioprinting technologies, because cells are always maintained and protected in an aqueous environment during the printing process [40]. The presence of such a water-based environment also allows the printer to freely deliver from single cell to multiple cells, by simply regulating the bioink concentration and the printed patterns.

Ink development is considered as one of the most challenging aspects in the bioprinting process. An ideal bioink should be highly biocompatible to hold live cells, mechanically stable after printing, and it should provide high resolution during printing [51]. Other important requirements for selecting a bioink for 3D bioprinting are permeability to oxygen and other gases, nutrients, and metabolic wastes, low cost, industrial scalability, and immunological compatibility [52].

The bioinks most commonly used in the 3D bioprinting are cell-laden hydrogels, decellularized extracellular matrix (ECM)-based solutions, and cell suspensions [53]. Hydrogels are hydrated networks of crosslinked polymers, in which cells can be encapsulated in 3D when the hydrogel undergoes gelation [54]. Over time, the scaffold biomaterial biodegrades and is replaced by cell-secreted ECM proteins; therefore, the encapsulated live cells grow occupying the space to form predesigned tissue structures [52]. These cell-laden hydrogel bioinks can be natural polymers, such as agarose, alginate, chitosan, collagen, gelatin, fibrin, and hyaluronic acid (HA); synthetic, such as pluronic and polyethylene glycol (PEG); or a combination of both. In any case, all hydrogel formulations require printing of a polymer solution followed by subsequent physical or chemical crosslinking [53]. Hydrogels are particularly attractive not only for their ability to accommodate live cells, but also for their adjustable chemical, mechanical and biodegradation properties, and the good resolution that can be achieved during printing [52]. Furthermore, hydrogels show low cytotoxicity and structural similarity to the native ECM. For cardiovascular bioprinting, soft bioinks can recapitulate the native elastic modulus, reliably mimicking the native physical properties of cardiac tissues and vasculature in the body.

Another bioink option is decellularization of allogenic or xenogenic tissues, which contain a variety of proteins, proteoglycans, and glycoproteins [48]. The use of decellularized ECM as bioinks offers the possibility to recreate more complex biologically and biochemically microenvironments, that mimic tissue specific ECM composition or resident cytokines [55]. Decellularized ECM-based bioinks are produced from the tissue of interest by removing the cells by sequential physical, chemical and enzymatic treatments while preserving an intact ECM [56]. The obtained ECM is then crushed into a powder and dissolved in a buffer solution to formulate the bioink for 3D printing. A polymer could be used as carrier to increase the solubility, to modify the viscosity, or to induce or increase post-crosslinking of the bioink [53]. Although decellularized ECM bioinks provide novel opportunities to fabricate tissue specific constructs, the decellularization process requires multiple steps, making it a costly approach. In addition, the use of decellularized ECM raises other concerns that must be addressed, such as immunogenicity and toxicity [57,58].

Other bioink materials recently explored in 3D bioprinting are cell aggregates. In cell aggregate configurations, the living cells are printed directly in a process resembling the normal embryonic growth. These approaches are emerging as an alternative to scaffold-based bioprinting [59]. One of the main advantages of the scaffold-free bioprinting is that problems related to the biomaterials’ compatibility are avoided; on the other hand, cell–cell interactions play a crucial role in tissue formation. Another attractive property of scaffold-free bioprinting is its efficiency, as the speed can be comparable or even higher than other forms of bioprinting [60]. Nevertheless, the time of pre-printing preparations tend to be longer when compared with scaffolds-based approaches. For this reason, scaffold-free-methods are preferable for smaller, cell-heterogeneous, matrix-poor tissues where the continuous intercellular communication is important. Cell aggregates can be classified into tissue spheroids, cell pellets, and tissue strands [61]. Tissue-spheroids are 3D organized clusters of cells generally 200–400 μm in diameter that, once placed near each other, fuse into a living material thanks to surface tension forces [62]. These spheroids possess the ability to organize as organoids in native tissue, and 3D bioprinting can be used for their precise assembling [59]. Substantial body of evidence has indicated that preformed cell spheroids have been assembled in cartilage, bone, and cardiac muscle-like constructs [59].

Despite the complexity of the cardiac tissue, 3D bioprinting is emerging as one of the most advanced techniques for creating cardiovascular implants possessing biomimetic features that recapitulate both the native physiochemical and biomechanical characteristics of the cardiovascular system. In the following paragraphs, we present some of the 3D bioprinting strategies used for fabricating functional cardiovascular tissues, including myocardium, heart tissue patches, and heart valves.

### 4.1. 3D Bioprinting of Functional Myocardium

The myocardium is the thick intermediate muscular layer of the heart wall responsible for contraction and relaxation of the heart [63]. The myocardium contains 2–4 billion cardiomyocytes (CMs), that account for roughly 75% of the heart volume, although they represent only about 33% of the total cell number [64]. CMs associate with nonmyocyte cells, that is endothelial cells (ECs), smooth muscle cells (SMCs), and fibroblasts (FBs), generating an intricately organized 3D structure.

**Figure 5:**
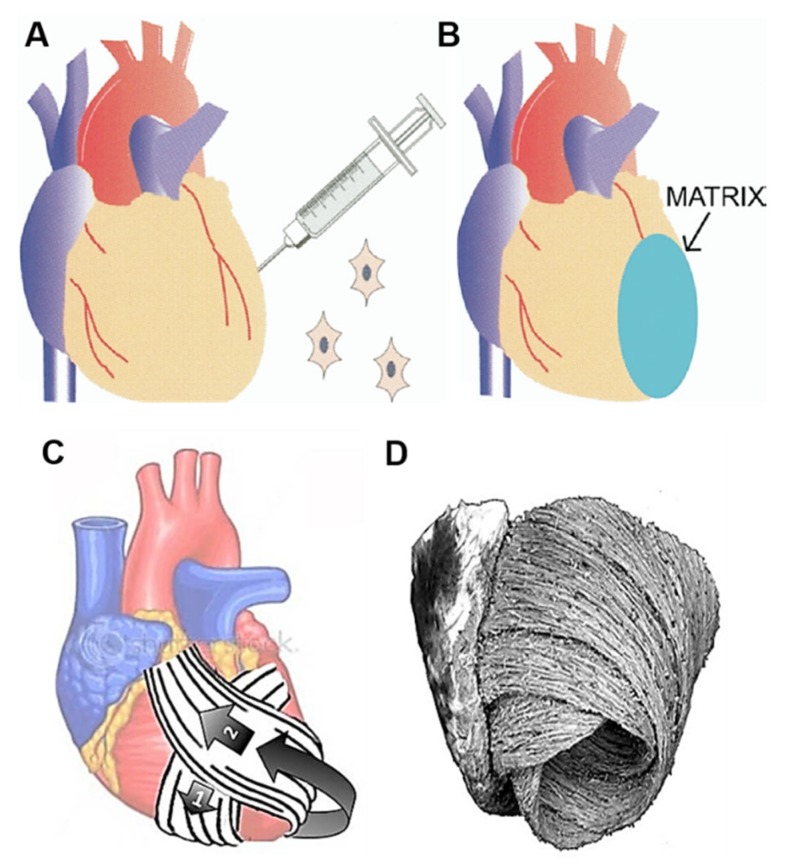
Schematic representation of the surgical procedure combining (**A**) the intra-infarct implantation of bone marrow cells (BMC) followed by (**B**) the fixation of a BMC-seeded collagen matrix onto the epicardial surface. Reprinted from Chachques et al. [68]. Copyright © 2020 The Society of Thoracic Surgeons. (**C**) Cardiowrap bioprostheses with helical loops, that follow (**D**) the anatomical heart configuration (native muscular ventricular bands). Modified from Chachques et al. [69]. Copyright © 2020 Informa UK Limited.

Most causes of heart failure are attributable to CMs deficiencies, and aging itself is associated with the loss of about 1 g of myocardium per year in the absence of specific heart disease [65]. CMs have a limited regenerative capacity, and during the progression of heart failure, also the cardiac ECM is modified and replaced by scar tissue [66]. Therefore, combining procedures aiming at regenerating both the CMs population and the ECM could improve the effectiveness of cellular therapy. In effect, over the years several tissue engineering strategies have been employed to create new myocardium that is electrically and mechanically integrated into the heart. The first successful contractile myocardium was fabricated in the late 1990s using chick embryonic CMs seeded onto a collagen scaffold [67]. Over the years, other myocardial tissue engineering procedures have been successfully explored to replace ECM in patients with ischemic heart disease or heart failure. For example, in the study of Chachques and colleagues, a biodegradable 3D collagen type I matrix was seeded with bone marrow cells (BMC) (250 ± 28 × 10^6^ cells), then grafted on the infarcted LV of 10 patients with chronic ischemic heart disease following autologous implantation of BMC (250 ± 28 × 10^6^ cells) on the scarred area (Figure 5A,B) [68]. Long-term results showed that a combined cell transplantation and matrix scaffolds approach offer further benefits with respect to cell therapy alone. Apart from being considered feasible and safe, this tissue-engineered approach improved the efficiency of cellular cardiomyoplasty. In addition, the cell-seeded collagen matrix allowed to normalize cardiac wall stress in injured regions, thus limiting ventricular remodeling and improving diastolic function. Subsequent studies combined the use of biodegradable cell-seeded hybrid scaffolds with synthetic mesh wrap devices for the creation of bioartificial myocardium and cardiowrap bioprostheses for ventricular support and myocardial repair (Figure 5C) [69,70]. In advanced heart failure patients, having large dilated ventricles, complete cardiac wrapping is associated with bioprosthetic helical myocardial bands, that follow the anatomical heart configuration of native muscular ventricular bands (Figure 5D). The role of such cardiopatches, complete wrap bioprostheses and helical myocardial bands is to reduce size and fibrosis of infarct scar, limit ventricular spherical dilatation, and recover elliptical LV shape. This positive chamber remodeling should contribute to improve diastolic filling and myocardial conditions by angiogenic, antiapoptotic and regenerative mechanisms. Preclinical studies in 18 sheep demonstrated that a cardiopatch manufactured using porous elastomeric polycaprolactone (PCL) 3D membrane filled with peptide hydrogel and stem cells improves myocardial infarct scars [71]. PCL elastomer was chosen for its good mechanical properties, namely flexibility and adaptation to curved surfaces, such as those of ventricles. The elastomeric membrane externally covered the heart surface, having advantageous restraining effects on ventricle dilation. In addition, a capillary network developed between the inserted scaffold and the heart.

**Figure 6:**
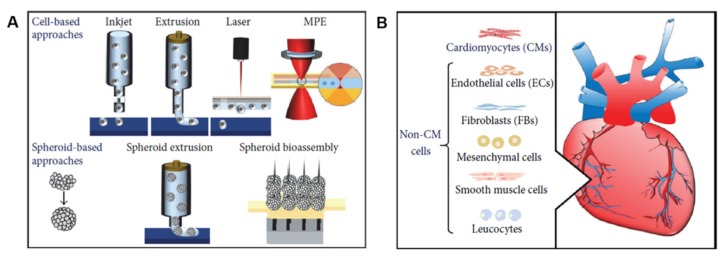
Schematic representation of 3D bioprinting of the myocardium, showing (**A**) the methods and (**B**) the cell types. Reprinted from Ong et al. [62]. Copyright © 2020 Chin Siang Ong et al.

These works provided the input for the development of scaffold-based 3D bioprinting works (Figure 6A). The bioinks mostly used to 3D bioprint myocardial tissue include alginate, collagen, gelatin, HA, and decellularized ECM [62]. Not only the choice of a suitable matrix, but also an appropriate cell source is critical for an effective cardiac regenerative therapy (Figure 6B). In this context, cardiac progenitor cells (CPCs) seem to be a promising cell population due to their natural differentiation potential to the cardiac lineages [72]. Nevertheless, during the years, other cell populations different from CMs have been used for brioprinting cardiac tissue models, including embryonic stem cells (ESCs), mesenchymal stem cells (MSCs), and induced pluripotent stem cells (iPSCs). The advantages in using these cell types is that most of them is produced pre-clinically and is safe and effective in clinical practice [48].

**Figure 7:**
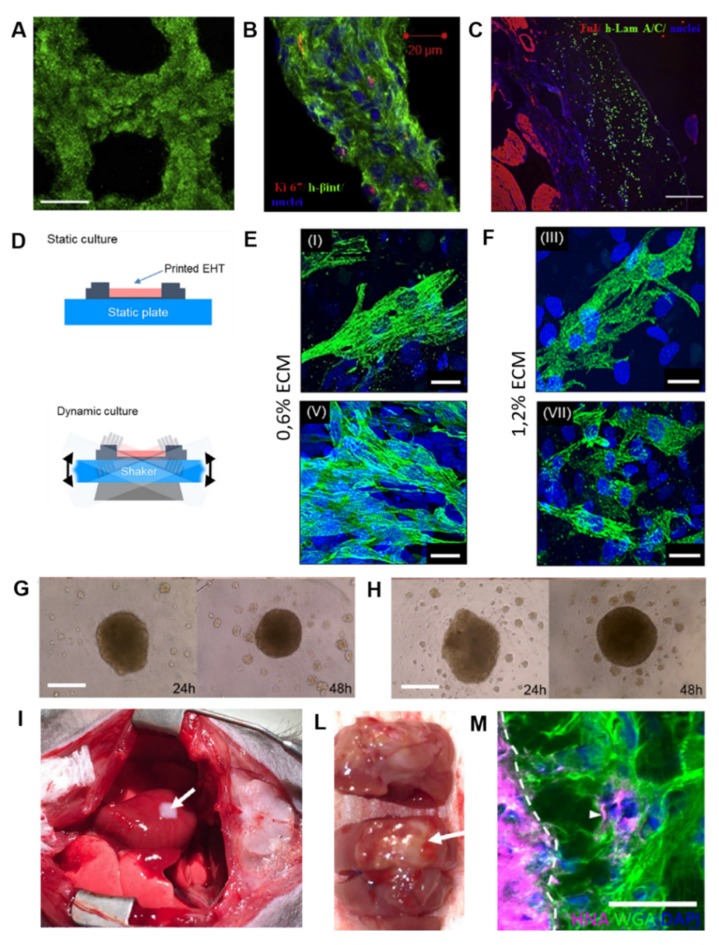
Examples of 3D bioprinting of myocardium using (**A**–**C**) cell-laden scaffolds, (**D**–**F**) decellularized heart ECM, and (**G**–**M**) cell-free scaffolds. (**A**) Human CMPCs 3D bioprinted in a scaffold made of HA and gelatin are alive (green) 2 hours after printing, (**B**) express the proliferation marker Ki-67 (red) after 7 days in culture, and (**C**) are visible in infarcted mice 4 weeks after transplantation. Reprinted from Gaetani et al. [73]. Copyright © 2020 Elsevier Ltd. (**D**) Schematic representation of 3D bioprinted cardiac decellularized ECM cultured under static and dynamic conditions. (**E**) confocal microscopy images of immunostaining for cardiac TnT (green) synthesized by CMs in 0.6%, and (**F**) 1.2% ECM cultured statically and dynamically for 14 days. Reprinted from Das et al. [84]. Copyright © 2020 Acta Materialia Inc. Cardiospheres form in 24 hours and start beating in 48 hours when iPSCs-CMs:FBs:ECs were co-cultured at (**G**) 70:15:15 or (**H**) 45:45:10 ratio. (**I**) Transplantation of 3D bioprinted cardiac patches (iPSCs-CMs:FBs:ECs 70:15:15) onto the anterior surface of the rat heart. (**L**) Anterior aspect of the heart explanted 1 week after implantation. (**M**) Confocal microscopy images of immunostaining for human nuclear antigen (HNA) (magenta), wheat germ agglutinin (WGA) (green), and DAPI (blue) showing the presence of human cells (white arrows) in native rat myocardium. White dotted line demarcates the cardiac patch (left) from the native rat myocardium (right). Reprinted from Ong et al. [88]. Copyright © 2020 Springer Nature.

Gaetani and coworkers used the method of pressure-based extrusion to bioprint a patch of human cardiac-derived cardiomyocyte progenitor cells (CMPCs) (30 × 10^6^ cells/mL) on a scaffold made from alginate, which was characterized by precise pore size and microstructure [66]. The authors demonstrated the cardiogenic potential of this patch, determined by mRNA upregulation of the early cardiac transcription factors NK2 homeobox 5 (Nkx2.5), Gata-4, myocyte enhancement factor 2C (Mef-2c), and of the late cardiac marker Troponin T (TnT) by these cells 7 days post-printing. Furthermore, the printed cells were able to migrate from the alginate matrix to a matrigel layer, forming tubular like structures. Few years later, the same group bioprinted a myocardial patch consisting of human CMPCs (30 × 10^6^ cells/mL) suspended in a scaffold made of HA and gelatin, then treated 10–12 weeks aged infarcted mice [73]. After 4 weeks from implantation, the scaffold proved to be an excellent vehicle to support cell survival, engraftment, and differentiation, finally improving mouse cardiac function (Figure 7A–C).

In order to bioprint 3D functional cardiac tissue constructs with improved conductive properties, Zhu and coworkers recently developed an attractive bioink containing an electrically conductive component [74]. To do this, the authors incorporated gold nanorods (GNRs) into a CMs- and cardiac FBs-laden hydrogel (1:1.5 × 10^6^ cells/mL), consisting of gelatin methacrylate (GelMA) and alginate pre-polymer solutions. GelMA is a degradable hydrogel composed of gelatin, which is the denatured form of collagens, that has been tunably substituted with methacryloyl groups [53]. In addition, the presence of integrin-binding motifs and matrix metalloproteinase(MMPs)-sensitive groups make GelMA a biomaterial highly bioactive [75]. The advantage of using alginate, instead, is represented by its ability to maintain the viscosity of the bioink for a long period of time at a room temperature [76]. After 5 days of culture, both the CMs and cardiac FBs were successfully embedded within the GelMA/alginate bioprinted construct loaded with GNRs, and displayed good cell spreading [74]. One week later, the two cell populations entirely colonized the printed construct, forming a uniform and interconnected tissue layer. Compared to the constructs without GNRs, the presence of a conductive nanomaterial in the GelMA/alginate bioink further improved the electrical propagation between adjacent CMs, as evidenced by higher expression of the gap junction protein Cx-43 after 14 days and higher synchronized contraction of the bioprinted construct.

An important challenge in the 3D bioprinting technology remains the vascularization of constructs, that is the ability of building in vessels that are capable of anastomosing with host vessels following implantation [77]. With the aim to construct a stable vasculature, Gaebel and colleagues used a polyester urethane urea (PEUU) cardiac patch for bioprinting two types of human cells, ECs (4 × 10^6^ cells) and MSCs (2 × 10^6^ cells), in a defined arrangement mimicking the vasculature [78]. It has been reported that co-implantation of vascular ECs and MSCs could enhance the stability of the neovascularization of ECs [79]. The authors reported better cell viability and increased vessel formation after 8 days of culture when compared to randomly seeded patches. However, the most interesting finding of this study was that the 3D bioprinted cardiac patch was able to promote the creation of a vascular network 8 weeks after implantation in infarcted rat hearts, enhancing vessel formation also in the border zone of myocardial infarction. Furthermore, significant improvements of heart function were observed following implantation of the bioprinted constructs in the infarcted area.

In some other recent studies, bioinks derived from cardiac decellularized ECM have been developed for 3D bioprinting and appeared to be promising biomaterials in the repair of myocardial dysfunction and for the delivery of stem cells. For example, Pati and colleagues successfully decellularized the LV of porcine heart ECM with a combination of physical, chemical, and enzymatic processes [80,81]. The resulting heart decellularized ECM possessed levels of collagen and glycosaminoglycans similar to those of the native ECM, thus providing a viable environment for 3D bioprinting. By using a multi-head tissue/organ building system, the authors printed the obtained ECM with rat myoblast cells, producing a tissue that had a myocardial-like organization. The 3D bioprinted constructs further supported differentiation and maturation of encapsulated cells, as demonstrated by the expression level of the cardiac-specific genes fast myosin heavy chain (Myh6) and alpha-sarcomeric actinin (Actn1) during 14 days study-period. In agreement with gene expression results, immunofluorescence analysis confirmed the presence of Myh6 in the cell-printed constructs. Jang and collaborators produced a heart decellularized ECM from the LV of 6-month-old Korea domestic pigs [55]. They used three different bioink formulations to develop pre-vascularized stem cell patch. In detail, 2 layers of PCL were made up to provide mechanical support. Then, bioink I, containing 5 × 10^6^ human CPCs/mL, and bioink II, containing 5 × 10^6^ human MSCs/mL supplemented with 10 µg/mL vascular endothelial growth factor (VEGF), were alternatively printed on the PCL supporting layer. After printing, vitamin B2 was used for the crosslinking process by exposition to ultraviolet radiation. The result of this dual stem cell 3D printed structure was a tissue with stiffness similar to native myocardium, in which the cells were able to proliferate and differentiate. In addition, the developed stem cell patch promoted strong vascularization and tissue matrix formation when implanted for 28 days into 7-weeks-old balb/c nude mice. Another interesting example of 3D bioprinting involving two different stem cell populations comes from the recent work of Park and colleagues, who employed a multipronged approach for simultaneously restoring cardiac function and vessel formation in infarcted rat hearts [82]. To do this, they intramyocardially injected CMs derived from human iPSCs (iPSCs-CMs) (1 × 10^6^ cells/rat), and epicardially implanted human MSCs-loaded patch (MSCs-PA) (1 × 10^6^ cells/mL) generated from pig heart decellularized ECM. Epicardial patch carrying human MSCs improved vascular regeneration through prolonged secretion of angiogenic paracrine factors, but more importantly it promoted the engraftment, viability and maturation of the injected human iPSC-CMs, ultimately leading to restoration of cardiac function in infarcted-induced hearts.

Another group combined cardiac decellularized ECM hydrogel with GelMA and pediatric human CPCs (3 × 10^6^ cells/mL) to print a patch to be used for pediatric patients suffering from right-ventricular (RV) failure or for adult myocardial dysfunction [83]. The presence of GelMA allowed for printability of the CPCs/cardiac ECM bioink through hydrogel polymerization via cooling to 10 °C, followed by white light radical polymerization and incubation at physiological temperatures. The authors demonstrated that the human CPCs cells in GelMA-cardiac ECM patches improved cardiogenic differentiation and angiogenic potential at 3 and 7 days when compared to cells in GelMA-only patches. In vivo, the printed GelMA-cardiac ECM patches remained attached to rat hearts epicardially, and vessels were formed after 14 days, indicating their integration with the native myocardium [83]. The importance of cardiac ECM as a component of bioinks was also underlined by Das and colleagues, who created bioinks with porcine heart tissue-derived ECM or collagen for encapsulating neonatal rat CMs (2 × 10^7^ cells/mL) using an extrusion-based 3D bioprinter [84]. The patches were then cultured for a month in dynamic or static conditions with the aim to evaluate the structural arrangement of CMs and their subsequent gene expression. From a molecular point of view, heart tissue-derived ECM cultured under dynamic conditions promoted enhanced expression of cardiac specific genes, like cardiac TnT, Myh6, and Actn2, compared with the static condition during the 14-day study period. This was also true for genes encoding the proteins responsible for cell–cell adhesion, cell-matrix interaction (integrins), formation of basement proteins (laminins), and guidance of matrix remodeling events (MMPs). Furthermore, microscopic images revealed that CMs in decellularized ECM cultured dynamically exhibited a more aligned, uniform, and rod-like structural arrangement of the cardiac regulatory protein TnT with sarcomeric integrity compared to the respective static counterpart (Figure 7D–F). The results of this work would indicate that both matrix and microenvironment can be decisive factors for cell–cell and cell–matrix interactions, thus influencing engineered heart tissue maturation.

In a very recent work by Noor and colleagues, a biopsy of omental tissue was taken from patients, then the cells were reprogrammed to become iPSCs and differentiated to CMs and ECs, whereas the ECM was processed into a personalized hydrogel serving as a bioink for 3D printing [85]. The bioinks were then 3D printed for generating vascularized patches (2 × 10^7^ iPSCs/mL and 2 × 10^7^ ECs/mL) and complex cellularized structures. Structure and function of the patch was evaluated in vitro, while cardiac cell morphology was assessed after transplantation in between two layers of rat omentum, revealing elongated CMs with massive actinin striation. This work has demonstrated for the first time the possibility to engineer vascularized cardiac patches that fully match the immunological, biochemical, and anatomical properties of any individual.

As mentioned before, scaffold-free techniques are being increasingly developed because problems associated with using biomaterials, such as immunogenicity, fibrous tissue formation, biomaterial degradation, toxicity of degradation products, are avoided. One of the first work is the one of Atmanli and Domian, who addressed the need of a scaffold using a microtextured polydimethylsiloxane (PDMS) stamp to guide the microarchitecture of murine ventricular progenitor cells (CVPs) printed by microcontact printing [86]. Although this experiment did not test the in vivo therapeutic properties of the bioprinted myocardium, it nonetheless represents a pivotal early step in scaffold-free 3D bioprinting of myocardial tissue. Later, Ong and coworkers 3D bioprinted cardiac patches by mixing cell cardiospheres (33 × 10^5^ cells/cardiosphere) composed of human iPSCs-CMs, FBs, and ECs at different ratios [87,88]. The assembling of cardiospheres resulted only when iPSCs or iPSC-CMs were co-cultured with at least 15% ECs or FBs. Patches of all cell ratios showed spontaneous beating, ventricular like action potential and uniform electrical conduction spontaneously within 3 days of bioprinting. By increasing FBs percentage, lower conduction velocities and longer action potential duration were recorded, suggesting some inhibition of electrical coupling of CMs by these cells. Nevertheless, after 1 week from implantation onto nude heart rats, all the bioprinted patches engrafted well on to the myocardium and were vascularized, indicating the regenerative potential of such a scaffold-free 3D bioprinting approach (Figure 7G–M). The applications of 3D bioprinting in the cardiac patches’ fabrication discussed in this paragraph are listed in Table 1.

### 4.2. 3D Bioprinting of Heart Valves

In addition to myocardial damage, heart valves disfunction represents another significant cause of heart failure [89]. Anatomically, heart valves consist of three leaflets and a root wall, mainly containing valve interstitial cells (VICs), SMCs, and valvular endothelial cells (VECs) [46]. These cells populate the valves in precise spatial locations having different flexure strength and stiffness [90]. The leaflets are indeed tri-layer structures, each characterized by unique ECM composition: the collagen-rich fibrosa layer, the intermediate proteoglycan-rich spongiosa, and the elastin-rich ventricularis [91]. These three layers continuously flaps, resulting in a closure and opening of the valve, and are subjected to shear stress and periodic loading and unloading. The roots, on the other hand, are annular structures providing support to the leaflet and serving as a base to be integrated with the major blood vessels of the heart [92]. In valve heart diseases, the valves become either too contracted to open-up entirely or incapable to close effectively. Currently, valve replacement surgeries are the only option for the vast majority of patients; these strategies usually employ mechanical or biological prosthetic valves [93]. Mechanical valves have high durability; however, the thrombogenicity of such valves is a major concern, forcing patients to take blood-thinning drugs for the rest of their lives. On the other hand, biological prosthetic valves suffer from other complications, including immune rejection and degeneration over time, thus requiring a possible reoperation after 10–20 years [93].

3D heart valve bioprinting has been explored as an alternative technology to traditional mechanical or biological prosthetic valve replacements, and also possesses further benefits over standard tissue engineering methods [94]. The advantages offered by 3D bioprinting include the possibility to generate a mechanically heterogeneous structure with spatial control of valve cells, therefore accurately replicating the complex biomimetic architecture of heart valves. Hydrogels are promising scaffold materials for bioprinting heart valves, due to their high physicochemical and mechanical tunability, and permeability to nutrients and waste for encapsulated cells. In addition, hydrogels can be designed to mimic biological properties of soft tissue heart valve scaffolds with high spatial precision [95,96]. Nonetheless, despite considerable efforts have been made in this field, it is still challenging to achieve completely controlled regional and spatial composition to bioprint the complex 3D structure of heart valves. Below, we report some examples of the most recent attempts of heart valves 3D bioprinting described in the literature.

**Figure 8:**
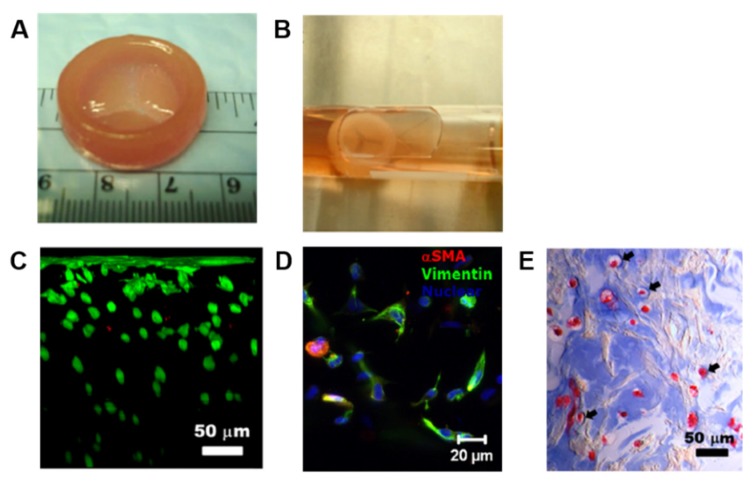
Example of 3D bioprinting of heart valve conduit with encapsulation of human aortic VICs (HAVICs) within the leaflets. (**A**) The valve conduit bioprinted using 4% w/v Me-HA/10% w/v Me-Gel hydrogels has inner diameter of 20 mm, outer diameter of 26 mm, height of 8 mm for valve root, and three leaflets with radius of 10 mm. (**B**) The bioprinted valve conduit shows an intact structure after photo-crosslinking and 7 days of static culture. (**C**) Cross-sectional view of Live/Dead image showing that nearly all encapsulated cells are alive from the surface to more than 300 μm below the surface. (**D**) Immunofluorescence images of the encapsulated HAVICs showing positivity for αSMA (red), vimentin (green), and nuclei (blue) after 7 days of culture. (**E**) Masson’s Trichrome staining of bioprinted leaflets showing that more intense blue color is found around the encapsulated HAVICs, indicating the newly deposition of collagen. Reprinted from Duan et al. [100]. Copyright © 2020 Acta Materialia Inc.

Duan and colleagues experienced different types of hydrogel bioinks for creating 3D bioprinted valves. In one of their study, a 3D bioprinted aortic valve conduit was created by encapsulating human aortic root SMCs and porcine aortic VICs (1 × 10^7^ cells/mL) in alginate/gelatin hydrogels (0.05 and 0.06 g/mL, respectively), using a dual syringe system to mimic the structure of valve root and leaflet, respectively [97]. Both cell types showed high cell viability after encapsulation within the hydrogel discs, being over 80% after 7 days from printing. Furthermore, encapsulated SMCs expressed elevated alpha-smooth muscle actin (αSMA), a contractile filament protein, whereas VICs expressed elevated vimentin, a protein typically present in FBs and other mesenchymal cells [98,99]. In a subsequent work, the same authors used human aortic VICs (HAVICs) (5 × 10^6^ cells/mL) suspended in methacrylated-HA (Me-HA)/methacrylated-gelatin (Me-Gel) hybrid hydrogel solutions (4% w/v of Me-HA, and 6%, 10%, 12% w/v of Me-Gel) to bioprint a tri-leaflet valve [100]. In detail, the heart valve root was printed with acellular hydrogel, while the leaflet was printed with the Me-HA/Me-Gel hydrogels suspended with HAVICs. Cells in all the hydrogel formulations maintained a post-printing viability of over 90% after 7 days, without significant difference in proliferation rate. By increasing Me-Gel concentration resulted in lower stiffness and higher viscosity, facilitating cell spreading, and better maintaining HAVICs fibroblastic phenotype. Histological and biomolecular analysis showed significant expression of αSMA, vimentin, periostin, and collagen type I, demonstrating that the cells proliferated normally post-printing and secreted their own ECM (Figure 8). However, both the studies described here were short-term works (7 days), and the printed constructs did not completely fulfil the mechanical performance of native valve tissue.

In the 3D bioprinting process, a high degree of geometric control and shape fidelity of an hydrogel construct can be achieved by using photo-crosslinking [101]. Light exposure is generally sufficient to solidify the hydrogel structure, and it can be performed during printing or post-fabrication of the whole construct [102]. Kang and colleagues tested several parameter combinations of photoinitiator type and concentration, and light intensity for optimizing cell viability during 3D bioprinting in a mixture of methacrylated gelatin/poly-ethylene glycol diacrylate/alginate (MEGEL/PEGDA/alginate) [102]. The two compared photoinitiators were Irgacure 2959 and VA086: Irgacure 2959 was chosen because it is one of the most widely used in contact with cells, whereas VA086 has been reported to be less cytotoxic than Irgacure [103,104,105]. As cell source, the authors used HAVICs, human aortic valve sinus smooth muscle cells (HASSMCs), and human adipose derived mesenchymal stem cells (HADMSCs) (2.5 × 10^6^ cells/mL). The encapsulated cell population that responded better to increased concentrations of photoinitiator was the HADMSCs. When using 0.25–1.0% w/v VA086, 0.025–0.1% w/v Irgacure 2959, and 365 nm light intensity 2–136 mW/cm^2^ for encapsulating cells in the the MEGEL/PEGDA/alginate bioink, cell viability was 95% for HASSMCs, 93% for HAVICs, and 93% for HADMSCs. The major findings of this work were the identification of different parameters that can be combined for optimizing the viability of different cell populations within 3D bioprinted hydrogels. Some positive examples of other cell types are coming from applications in neuroscience and neurosurgery, where cells grown in either two-dimensional (2D) or 3D systems offer exciting opportunities in both basic [106] and in concrete regenerative tasks [107,108].

3D bioprinting has been successfully used also for generating 3D models of calcific aortic valve disease (CAVD) recapitulating leaflet layer biomechanics [109]. As mentioned above, the healthy aortic valve (AV) is composed of three semilunar leaflets, each comprising three stacked layers with its own ECM composition [91]. With the progression of CAVD, the leaflets of the AV become fibrotic and calcify as their constituent cell population of VICs undergo myofibrogenic and osteogenic differentiation [91]. In their study, Van der Valk and colleagues bioprinted a model of CAVD encapsulating human VICs (10 × 10^6^ cells/mL) in GelMA/methacrylated HA (HAMA) hydrogels followed by UV crosslinking with the photoinitiator lithium phenyl-2,4,6-trimethylbenzoylphosphinate [109]. The 3D printed hydrogels with encapsulated VICs were then cultured with osteogenic factors for promoting the formation of microcalcifications. The exposition to osteogenic stimuli for 14 days effectively caused the formation of microcalcific nodules; nevertheless, negligible levels of apoptosis were measured in the encapsulated cells, thus suggesting that calcification was not related to cell death by apoptotic processes. In this study, the authors additionally quantified the compressive mechanical properties of each of the AV leaflet layers, finding that the ventricularis showed an intermediate Young’s modulus (26.9 kPa) between the fibrosa and the spongiosa layers (37.1 kPa and 15.4 kPa, respectively). The findings of this work have established for the first time a novel 3D model for the study of valvular mechanobiology and could also facilitate high-throughput drug screening for CAVD in a biologically-relevant environment. 3D bioprinting applications for the generation of heart valve models discussed in this paragraph are summarized in Table 2.

### 4.3. 3D Bioprinted Heart Tissue Patches for Drug Screening

An increasingly widespread opinion believes that 3D printing can offer great potential also in pharmaceutics for the discovery and development of new drugs, as well as for advances in drug delivery strategies [94]. Drug discovery represents the preclinical phase of the entire process, and it consists in testing thousands of compounds to select few candidates with possible beneficial effects against the clinical target diseases. The drug development phase involves further validation of the selected drug in terms of absorption, distribution, metabolism, excretion (ADME), toxicity, dosage, and treatment modalities, as well as interaction with other molecular compounds [110]. Drug development is a multistep process that involves phase I-III clinical trials, review and approval by the Food and Drug Administration (FDA), phase IV clinical trials, followed by launch in the market, and post-market surveillance by FDA.

About 94% of the drugs that passed the preclinical trials fail in the clinical phase [111]. It is believed that this is mainly attributable to inadequate screening in preclinical trials, most of which are still performed on traditional 2D monolayer culture systems that miss the native 3D extracellular microenvironment [112]. Furthermore, the efficacy and toxicity of drugs evaluated in animal studies do not always predict the response in human patients [113]. For example, during both the preclinical and clinical stages of drug development, cardiotoxicity remains a major cause of failure, thus representing the primary reason for the retraction of pharmaceuticals from the market [114]. The use of 3D engineered tissue platforms could overcome these problems, and 3D bioprinting, in particular, could play a key role in both the drug discovery and development phases. 3D bioprinted tissues could produce results similar to those obtained in in vivo tests, thus representing valuable platforms for conducting drug toxicity analysis in vitro.

**Figure 9:**
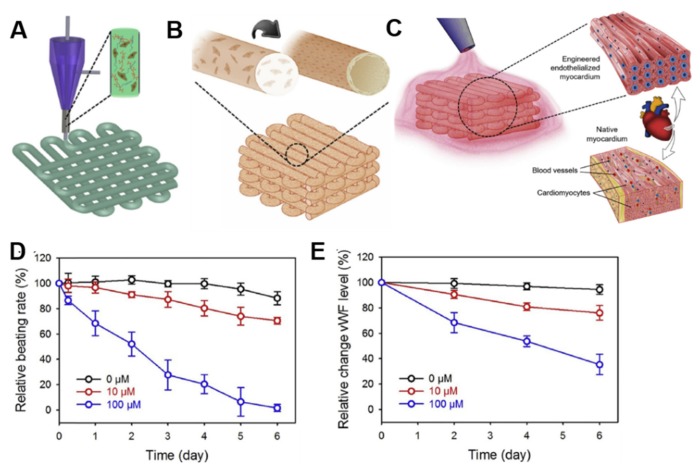
Example of 3D bioprinted heart tissue patches used for drug screening. (**A**) Schematic representation of the extrusion-based 3D bioprinting system used to generate microfibrous alginate/gelatin methacrylate (GelMA) scaffolds encapsulating endothelial cells (ECs), that (**B**) in approximatively 2 weeks form a vascular bed through migration of cells to the peripheries of the microfibers. (**C**) Cardiomyocytes (CMs) are then seeded into the interstitial space of the endothelialized scaffold. The doxorubicin dose-concentration response is evaluated as (**D**) relative beating rate of CMs and (**E**) relative expression levels of von Willebrand factor (vWF) in ECs. Reprinted from Zhang et al. [116]. Copyright © 2020 Elsevier Ltd.

In the cardiovascular field, most of the efforts toward developing drug discovery and screening platforms have focused on recreating microtissues of the left ventricular myocardium, the site of most cardiac pathologies and the primary pumping chamber of the heart [115]. For example, Zhang and colleagues developed a novel hybrid strategy based on 3D bioprinting to engineer endothelialized myocardial tissues [116]. In their work, the authors directly encapsulated ECs (1 × 10^7^ cells/mL) within an alginate/GelMA bioink through a combination of extrusion and photocuring processes (Figure 9A). In approximatively 2 weeks of culture, the ECs gradually migrated toward the microfiber peripheries, organizing in a confluent layer of endothelium, and formed a pattern resembling the blood vessel walls (Figure 9B). This bioprinted microfiber scaffold was then seeded with rat-derived CMs (1 × 10^6^ cells/mL) (Figure 9C). The CMs adhered and spread on the surface of the microfibers across the entire thickness of the scaffolds, and strongly expressed the contractile Actn1 protein and the intercellular conductive connexin-43, as demonstrated by immunostaining analysis. In addition, the CMs-populated constructs started beating spontaneously and synchronously after 48 hours of culture. In order to construct the endothelialized-heart-on-a-chip device and evaluate cardiovascular drug toxicity, a microfluidic perfusion bioreactor was generated. The endothelialized microtissue was tested with the common anti-cancer drug doxorubicin, which demonstrated dose-dependent effects towards both CMs and ECs. In particular, the beating rate of the CMs decreased to 70.5% and 1.62%, and the levels of von Willebrand factor (vWF) secreted by the ECs were reduced to 76.0% and 35.3% 6 days after exposure to 10 μM and 100 μM doxorubicin, respectively (Figure 9D,E). In the same study, the authors adopted a similar fabrication method and drug toxicity test on endothelialized human iPSCs-derived CMs microtissue, with results that were comparable to those observed in the rat-derived microtissue, thus suggesting potential translational for personalized drug screening.

More recently, Lind and coworkers designed cardiac microphysiological devices via multi-material 3D bioprinting by sequentially using six functional bioinks based on highly conductance, piezoresistive, and biocompatible soft materials [117]. After printing, the devices were seeded with human iPSCs-CMs (220 K/cm^2^), which self-assembled into laminar tissues mimicking the structure of the native heart. Such a fabricated system allowed the authors to perform dose response studies of cardiac drugs that influence contraction strength or beat rate directly inside a cell incubator. In particular, the L-type calcium channel blocker verapamil and the β-adrenergic agonist isoproterenol were investigated. The engineered microtissue displayed inotropic responses to verapamil and isoproterenol, comparable to data obtained from engineered 3D neonatal rat ventricular myocardial tissues and isolated postnatal whole rat hearts, therefore demonstrating the potential of this model as a drug screening platform.

In another study, Wang and colleagues 3D bioprinted a functional cardiac tissue mimicking the structural, physiological, and functional features of native myocardium [118]. The authors used a fibrin-based composite hydrogel as bioink to print primary CMs (10 × 10^6^ cells/mL) isolated from infant rat hearts. Bioprinted cardiac tissue constructs exhibited spontaneous synchronous contraction in culture, and positivity to Actn1 and connexin 43, indicating the generation of uniformly aligned and electromechanically coupled cardiac cells. The authors then evaluated physiologic responses of these bioprinted cardiac tissues to known cardiac drugs, the androgen agonist epinephrine and the androgen antagonist carbachol after 3 weeks of culture. Calcium imaging analysis was used to quantify spontaneous beating of bioprinted cardiac tissues, revealing that epinephrine increased the beating frequency from 80 to 110 beats per minute (BPM), whereas carbachol decreased the beating frequency to 40 BPM. Interestingly, the authors demonstrated the reversible effects of these drugs when these were removed from the bioprinted cardiac tissues, thus indicating their effectiveness in testing the physiological response of cardiac drugs.

As mentioned above, 3D bioprinting could be useful also for the development of drug delivery strategies [94]. Some current applications of 3D printing in drug delivery systems include implant surfaces (i.e., orthopedic implants) modified with drug-eluting solutions [119,120], medical devices (i.e., stent or catheter) containing and eluting drugs [121], and 3D printed delivery devices providing personalized drug release profiles [122]. 3D bioprinting applications for drug screening discussed in this paragraph are summarized in Table 3.

## 5. 3D Printing for Testing and Realizing New Heart Devices

**Figure 10:**
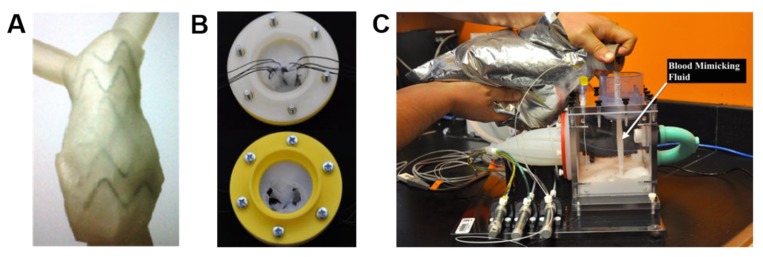
Examples of 3D printed models for device testing. (**A**) 3D printed right ventricular outflow tract (RVOT) model used for physical insertion of a stent-graft for assessing patient’s suitability for the device. Reprinted from Biglino et al. [124]. Copyright © 2020, Springer Nature. (**B**) Silicone 3D printed mitral valve (MV) incorporated into (**C**) a pulse-duplicator chamber filled with a blood mimicking fluid for hemodynamic testing. Reprinted from Mashari et al. [125]. Copyright © 2020 Elsevier Inc.

Another important application of 3D printing is the development and testing of medical devices. With regard to the cardiovascular field, one of the first studies was the one of Kalejs and von Segesser, who created an aortic root model made of common house-hold silicone, then started to use this model in in-vitro valved stents testing, integrating the aortic root in an artificial circulatory loop [123]. Biglino and coworkers, instead, imaged a tract of descending aorta of a 29-year-old volunteer 50 mm in length with MRI, then used the polyjet technique for printing models having different wall thickness (0.6, 0.7, 0.8, 1.0, and 1.5 mm) but constant internal diameter (15.5 mm) [124]. These models of descending aorta were then subjected to compliance tests through gradual increase and decrease of the internal volume in order to track pressure variations. Two critical cases of vascular anatomies were then selected for evaluating the practicability of the method, after the identification of a range of material’s distensibility. The first case concerned an adult patient affected by severe pulmonary regurgitation and dilated right ventricular outflow tract (RVOT), whereas the second case was about a pediatric patient with hypoplastic left heart syndrome (HLHS) and aortic coarctation (2.7 mm narrowing). Good anatomical finishing was qualitatively obtained for both models, suggesting their usefulness for device implantation and testing (Figure 10A). Later, Mashari and colleagues acquired TEE images of a patient subjected to a percutaneous MitraClip^®^ operation, which is a minimally invasive procedure to reduce the mitral regurgitation, for creating a 3D MV model [125]. Hemodynamic tests were then carried out on this 3D model by insertion in a pulse duplicator chamber loaded with a fluid mimicking the blood (Figure 10B). Paulsen and colleagues used a 3D printed left heart simulator to compare two different conduits for valve-spearing aortic root replacement [126]. The simulator was efficient to show the comparability of straight tubular and Valsalva grafts in terms of gross hemodynamic and coronary blood flow, however it revealed that the two conduits differ considerably in terms of biomechanics. 3D printing also resulted effective in realizing personalized occluders for the treatment of CHD using new biodegradable materials. As an example, Sun and coworkers produced a 3D printed biodegradable occluder made of a copolimer composed by poly-L lactic acid, trimethylene carbonate and glycolide [127]. Such a 3D printed model was then tested both in vitro and in vivo in a rabbit model, demonstrating good results in terms of safety, reliability and biocompatibility.

## 6. Regulatory Considerations and Commercialization of 3D Printed and 3D Bioprinted Products

The 3D printing industry is a rapidly expanding field, and it has been estimated that the global market for 3D printed devices will reach around $34.8 billion with a compound annual growth rate (CAGR) of 23.2% during the forecast period 2019–2024, while that of 3D bioprinting will be near $1.647 million with a CAGR of 20.4% by the end of 2024 [128,129]. For this reason, it is necessary that regulatory agencies around the world will soon try to establish quality standards before devices manufactured with 3D printing technologies become part of common clinical practice.

**Figure 11:**
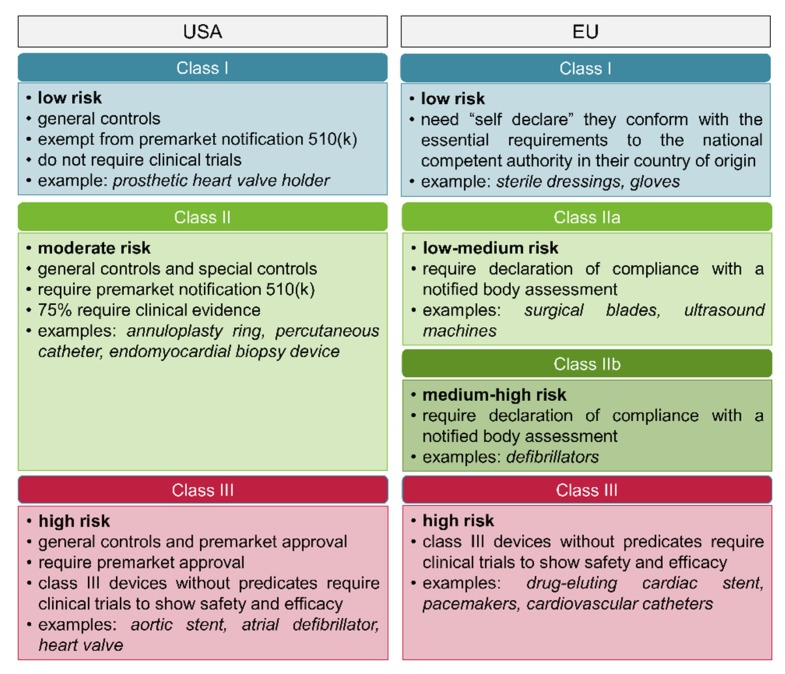
Classification of medical devices in the USA and in the EU.

Worldwide, the regulatory requirements for a given type of medicinal product depend strictly on its classification. In the USA, medicinal products are categorized as drugs, biologics, or medical devices [130]. Each type of product is regulated by a different center within the FDA: The center for drug evaluation and research (CDER), the center for biologics evaluation and research (CBER), or the center for devices and radiological health (CDRH), respectively. The FDA regulatory agency in the USA considers 3D printed devices in the same way to traditional medical devices, and therefore subject them to the same regulatory requirements and submission information [131]. The CDRH classifies medical devices into three classes, on the basis of the intended use of the device, the indications for use, and the risk level to patient or user (Figure 11) [131]. Medical devices of Class I have a minimal harm potential to the patient or user, require general controls without clinical trials and are exempt from the regulatory process, meaning there is no need for proof of safety or efficacy. Medical devices of Class II have a moderate risk to the patient or user, require both general and special controls, and most of them need also filing a premarket notification, known as 510 (k), for notifying the FDA the intent of marketing. A 510 (k) is obtained when the device proves to be safe, effective, and substantially equivalent to a device already legally marketed that is not subjected to premarket approval [131]. Finally, Class III includes those medical devices that support or sustain human life, are generally implanted, and thus require clinical trials or other evidences before the most rigorous premarket approval. If a Class III device presents only minor changes from an already approved device, named predicate device, it may not need the stringent premarket approval; on the contrary, all novel devices that do not have a predicate device are automatically classified as Class III devices [130,132].

In the EU, products are categorized as medicinal products (drugs or biologics) or medical devices, and they have their own unique regulatory processes [133]. With establishment of the EU, approval processes for medical devices followed a path of harmonization; nevertheless, medical device regulation does not fall solely to any one agency. Indeed, national competent authorities (NCAs) are responsible to designate and audit notified bodies (NBs), which are independent commercial organizations that contract with device manufacturers to supply these certifications for a fee [134]. NBs assess and assure conformity with requirements of the relevant European commission (EU) directives. Once NBs demonstrate the device meets requirements for conformity, the NBs issue a conformité européenne (CE) mark, and the device can be marketed in any EU member state [135]. Differently from the USA, medical devices in the EU are classified into four classes: class I, class IIa, class IIb, and class III (Figure 11) [136]. Class I are low risk medical devices, and only require a self-declaration of conformity by manufacturers without the involvement of NBs. In contrast, and similarly to what happens in the USA, medical devices of Classes IIa, IIb, and III require clinical and/or nonclinical evidence to support their approval.

**Figure 12:**
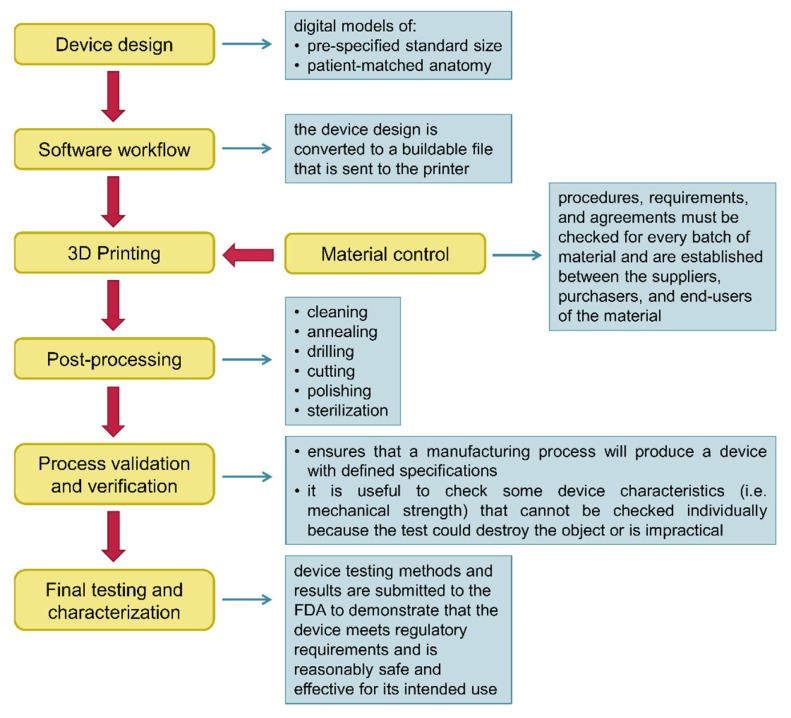
Technical workflow for manufacturing 3D printed medical devices.

In December 2017, the FDA released a guidance document entitled “Technical Considerations for Additive Manufactured Medical Devices”, with the aim to outline technical considerations for manufacturing 3D printed devices, and containing recommendations for testing and characterizing such devices [137]. As already discussed in a dedicated section of this review, the technical workflow for manufacturing 3D medical devices involve several steps (Figure 12). The aspects of 3D printing addressed in the guidance document focused on device design, materials control, printing and post-printing validation; examination of printing parameters; assessment of physical and mechanical characteristics of final devices; and biological and safety considerations of final devices in terms of cleaning, sterility, and biocompatibility [137]. It has to be remembered that 3D printed medical devices, like those traditionally manufactured, must meet quality system requirements across all the production process.

Over the years, more than 80 3D printed medical devices have been approved by the FDA’s center CDRH through 510(k) process [138]. Most of these devices are used in dental, orthopedic and cranio-maxillofacial fields (i.e., dental crowns, hearing aids, bone tether plates, skull plates, hip cups, spinal cages, facial implants, and screws), and are produced using powder bed fusion techniques (83%), stereolithography (12%), extrusion (3%), and jetting (2%) [138,139]. A postmarket analysis of the FDA’s approved devices, conducted on the manufacturer and user facility device experience (MAUDE) database, evidenced that the principal reasons for product-related adverse events were improper size or incompatibility with other components of the device (46%), device fracture or shaving (45%), incorrect device size for the patient (5%), implant wear (2%) and incorrect device implanted (2%) [138]. To be fair, we have to say that many of these adverse events are common to medical devices manufactured with the traditional techniques.

As for 3D printed products containing biological components, no defined regulations currently exist, since these products cannot be simply classified as medical device, biologic, or drug, but can rather be considered both medical devices and biologics [140]. Indeed, combination products are products comprising two or more regulated components, that is drug-device, device-biologic, drug-biologic or drug-device-biologic [141]. A combination product is for example one in which living cells (biologics) are combined with a synthetic scaffold matrix (medical device) that provides physically support for the growth of new tissue [133]. The legislation applied to these products depends on their classification, which is determined by several factors, mainly by the way in which the finished product achieves its intended medical purpose and by its primary mode of action (PMOA) [133]. In the USA, the office of combination product (OPC) assigns combination products to the most appropriate FDA center [142]. If the PMOA of a cell-based medicinal product is provided by the biological cellular constituent, then the product is regulated as biologics by CBER in the USA, and as combined advanced-therapy medicinal products (ATMPs) in the EU [140,142]. On the other hand, if the therapeutic effect derives from the device component, the combination product is considered a Class III medical device in the EU. Regarding 3D bioprinted products, their classification mainly depends on whether these devices are mass-produced or custom-made. In current practice, custom-made devices, which are manufactured in accordance with a specific prescription for a specific individual, are exempt from normal quality system requirements or conformity assessment requirements by most regulatory bodies in both in the USA and in the EU [143,144].

The technical workflow for 3D bioprinting medical device is similar to that seen for 3D printing, although additional steps are included in the manufacturing process, such as selection of a design approach, cell type, and biological material [138]. Consequently, additional technical considerations need to be taken into account respect to 3D printed medical devices. These include, for example, printing parameters (printing temperature, resolution, and speed) or material selection. Mechanical and physicochemical properties, biocompatibility, as well as vascularization and biological function of the finished product are other parameters to be evaluated. Although 3D bioprinting technologies are largely still in the research and development stage, urgent regulatory and manufacturing process considerations are required to meet the growing global interest and needs of such 3D printed products.

## 7. Current Limitations, Future Perspectives, and Conclusions

3D printing attracted remarkable attention of the scientific community due to its high versatility, and in recent years the technology has considerably expanded also to the cardiovascular medicine. 3D printing allows the complex cardiac anatomy to be accurately replicated using materials resembling human heart tissues. For this reason, 3D printed heart models could serve as an excellent tool in facilitating medical education, pre-operative planning, and communication between doctor and patients or their family. Nevertheless, cardiac surgeons and cardiologists agree with the fact that these 3D printed heart models should only be used to complement the current diagnostic tools. This is particularly true when creating a model for cardiovascular pre-surgical planning, where an error made during segmentation of an image data set could have devastating consequences. In addition, there is still no statistical data currently available to demonstrate the real clinical value of these 3D printed models. Another obstacle that limits diffusion and routine application of 3D printing in clinical practice is related to its high cost. Further investigations are needed to analyze the cost-benefit of 3D printing for creating anatomical models. Interestingly, a recent work demonstrated that 3D printed heart models produced with low cost materials (A$50) show efficiency and precision similar to models created with high cost materials (A$300) [6]. Another limitation to overcome in a near future is the availability of the 3D printing technology in situ; most research hospitals, indeed, have access to facilities through academic collaborators. Building a 3D printing laboratory would require planning in hardware, software and dedicated staff [145].

Currently, the most advanced application of 3D printing is 3D bioprinting, which has the great potential to engineer highly organized functional tissues and/or organs with complex geometries and tailored components for widespread applications, including transplantation, drug discovery and development, and disease modelling. The most ambitious goal of the 3D bioprinting technology, however, is the realization of customized devices for clinical applications. In the cardiovascular field, fabrication of 3D bioprinted functional tissues greatly depends on the availability of biomimetic materials recapitulating native heart ECM and electrical conductivity properties. Although several specialized 3D bioprinters have been developed to fabricate various types of 3D heart tissues, such as myocardium and heart valves, bioprinting technology still faces many technical challenges. Some of the main efforts focus on obtaining high resolution distribution of cells, while minimizing their loss and maximizing cell–cell interactions. The ideal bioprinter should have resolution in the submicron range to allow bioprinting of a matrix with an orientation able to direct the alignment of cardiovascular cells [146]. Achieving the proper cellular composition is another issue in cardiovascular 3D bioprinting [62]. As emerged in different studies discussed in the manuscript, alteration of the composition of nonmyocyte cells, such as FBs and ECs, strongly influences the function, vascularization and vitality of the 3D bioprinted cardiac tissue.

Another crucial aspect to consider in 3D bioprinting relates the development of an ideal cardiac bioink, possessing appropriate stiffness and cell microenvironment, and controllable degradation rate. At the time of this writing, hydrogels are among the most used bioprintable materials for cardiovascular applications, as they provide good support for cells. Nevertheless, hydrogels do not contain specific ECM proteins for certain cell types, thus not fully reflecting the native environment of the heart. In addition, although hydrogels encapsulate and confine cells, they limit cell–cell interactions, and do not allow to obtain the same high cell density as native tissues. Another important shortcoming is the instability of hydrogels during the bioprinting process: Increasing hydrogel concentration improves its mechanical properties, but simultaneously limits biological activities [147]. Use of decellularized heart ECM as bioink material represents an alternative solution in 3D bioprinting, although further optimization is still needed. Besides the fact that decellularization is a tedious process, the native ECM loses its mechanical integrity and some of the biochemical properties when homogenized, and toxic residues can remain after the decellularization process. In addition, cells seeded on the decellularized ECM produce MMPs that rapid degrade the bioink [147]. Despite the advancements in scaffold-free techniques, limitations still exist also in the use of tissue spheroids for 3D bioprinting. Some are technical difficulties related to the printing process: The nozzle used to load tissue spheroids should be large enough to hold the spheroids inside and allow extrusion without clogging, since these structures do not have a homogeneous size and can be easily deformed or broken. Furthermore, necrosis can occur in the core of tissue spheroids, but this can be overcome by proper vascularization. Considering that each type of bioink has its own advantages and disadvantages over others, a future winning strategy could involve the combination of bioinks composed of multiple and complementary biomaterials. This in turn would require the development of hybrid systems integrating different but compatible bioprinting technologies. At present, the majority of the 3D bioprinting techniques require post-processing of the printed product through crosslinking. In most cases, this step complicates the workflow and leads to a potential deterioration in the quality of the final product [148]. For these reasons, there is an urgent need to improve material design or the printing technology itself for preventing the post-printing step.

As discussed in the manuscript, another challenge in 3D bioprinting is the achievement of early vascularization of tissue constructs, which is fundamental for exchange of materials and oxygen supply, and consequently for improving vascular integration with the host cardiovascular system. Supply of oxygen to 3D printed tissue constructs could be addressed by generation of oxygen biomaterials that, together with conductive elastomers, could be the future of 3D bioprinted cardiac tissues. In addition, the development of 3D printed bioreactors that can integrate electrical and mechanical stimulation could represent another potential area for generating functional heart tissues [149].Advancements in 3D bioprinting technologies will also be dependent on the capability to accelerate large-scale tissue constructs production in a rapid manner. At the moment, only extrusion-based bioprinting has the potential to produce volumetric human-scale tissues but this method lacks speed and resolution. A final challenge in this field is the conduction of long-term studies for evaluating cell viability after printing, cell phenotypic changes and their biological functionality, as many works published so far are short-term studies. Long-term studies could better assess the biological safety and fidelity of constructs before clinical trials. In this context, immunogenicity of the 3D bioprinted construct is an issue that has to be addressed as well.

A future area of research is undoubtedly 4D printing, which essentially combines a 3D printing technique with smart materials that can respond to external stimuli, thereby reshaping or changing their function over time [150]. To do this, 4D printing employs hydrogels responsive to, i.e., temperature, pH, light, electric or magnetic field, or shape-memory polymers [151]. The most intriguing aspect of 4D bioprinting is the possibility to create sophisticated high resolution dynamic and animated structures, otherwise inaccessible with the current static 3D bioprinting techniques. Nonetheless, an aspect that should be carefully considered in 4D printing is the presence of a stimulus that may have a negative effect on living cells. At the moment, 4D printing of cardiac tissue is still in its early stages, but we believe that it can give the opportunity to generate dynamic structures better mimicking the types of tissue structures in vivo. It is hoped that in a near future these important major challenges and regulatory issues could be addressed to enable the translation of the technology to personalized therapeutic and pharmaceutical applications.

## Figures and Tables

**Table 1:** Applications of 3D bioprinting for production of cardiac patches.

Bioink	Cell (Concentration)	3D Bioprinting Technique	Significance	Ref.
alginate	human CMPCs ^1^ (30 × 10^6^/mL)	extrusion-based bioprinting	cells were able to migrate out of the alginate matrix and fully colonize a matrigel layer, forming tubular-like structures in vitro	[66]
HA ^2^ and gelatin	human CMPCs (30 × 10^6^/mL)	extrusion-based bioprinting	the scaffold was able to support cell survival, engraftment, and differentiation; in addition, it improved cardiac function after epicardial transplantation in a mouse model of myocardial infarction	[73]
GelMA ^3^ and alginate incorporating GNRs ^4^	CMs ^5^ (1 × 10^6^/mL) and cardiac FBs ^6^ (1.5 × 10^6^/mL)	extrusion-based bioprinting	the presence of a conductive nanomaterial (GNRs) into the hydrogel improved the electrical propagation between adjacent CMs, that finally resulted in a synchronized contraction of the bioprinted construct in vitro	[74]
PEUU ^7^	human ECs ^8^ (4 × 10^6^) and human MSCs ^9^ (2 × 10^6^)	laser-based bioprinting	co-implantation of ECs and MSCs in a defined printed pattern enhanced the vascularization of the construct and improved cardiac function after acute myocardial infarction in rats	[78]
decellularized ECM ^10^ from the LV ^11^ of porcine heart	rat myoblast cells (from 1 to 5 × 10^6^/mL)	extrusion-based bioprinting	the construct possessed a microarchitecture having a native-like organization	[80,81]
decellularized ECM from the LV of 6-month-old Korea domestic pig	human CPCs ^13^ (5 × 10^6^/mL) and human MSCs (5 × 10^6^/mL)	extrusion-based bioprinting	the use of two different bioink formulations, one containing CPCs and the other made of MSCs supplemented with VEGF ^14^, allowed for the development of pre-vascularized cardiac patch	[55]
decellularized ECM from the LV of porcine heart	CMs derived from human iPSCs ^15^ (1 × 10^6^/rat) and human MSCs (1 × 10^6^/mL)	extrusion-based bioprinting	the strategy of intramyocardially applying CMs derived from human iPSCs-CM and epicardially implanting a cardiac patch containing human MSCs significantly improved cardiac function and vessel formation in a rat model of myocardial infarction	[82]
decellularized ECM from porcine ventricular tissue combined with GelMA	pediatric human CPCs (3 × 10^6^/mL)	extrusion-based bioprinting	possibility of using the cardiac patch in pediatric patients suffering from RV ^16^ failure, or for treating adult myocardial dysfunction	[83]
decellularized ECM from the LV of porcine heart or collagen	neonatal rat CMs (2 × 10^7^/mL)	extrusion-based bioprinting	the culture conditions (dynamic versus static) are decisive factors for the structural arrangement of CMs, and affect gene expression and the related signaling pathways	[84]
decellularized human omental tissue	human iPSCs-CMs ^17^ (2 × 10^7^/mL) and ECs (2 × 10^7^/mL)	extrusion-based bioprinting	possibility of generating vascularized patches that fully match the immunological, biochemical and anatomical properties of any individual	[85]
scaffold-free	cardiospheres (33 × 10^5^ cells/cardiosphere) composed of human iPSCs-CMs, FBs and ECs at different ratios	3D bioprinting on a needle array	the biomaterial-free 3D printed cardiac patch produced from human iPSCs showed spontaneous beating, electrical integration of the cardiospheres, and in vivo engraftment and vascularization	[87,88]

^1^ cardiomyocytes progenitor cells; ^2^ hyaluronic acid; ^3^ gelatin methacrylate; ^4^ gold nanorods; ^5^ cardiomyocytes; ^6^ fibroblasts; ^7^ polyester urethane urea; ^8^ endothelial cells; ^9^ mesenchymal stem cells; ^10^ extracellular matrix; ^11^ left ventricle; ^13^ cardiac progenitor cells; ^14^ vascular endothelial growth factor; ^15^ induced pluripotent stem cells; ^16^ right ventricle; ^17^ cardiomyocytes derived from human induced pluripotent stem cells.

**Table 2:** Applications of 3D bioprinting for production of heart valves.

Bioink	Cell (Concentration)	3D Bioprinting Technique	Significance	Ref.
alginate/gelatin	human aortic root smooth muscle cells (SMCs) ^1^ (1 × 10^7^/mL) and porcine aortic valve interstitial cells (VICs) ^2^ (1 × 10^7^/mL)	extrusion-based bioprinting	the use of a dual syringe system, each containing a defined cell population (SMCs or VICs), allowed for the creation of a 3D printed aortic valve conduit complete of valve root and leaflet	[97]
Me-HA ^3^ and Me-Gel ^4^	HAVICs ^5^ (5 × 10^6^/mL)	extrusion-based bioprinting	a heart valve conduit was bioprinted with acellular root and three leaflets encapsulating HAVICs; by varying the concentration of the hydrogel formulations it was possible to modulate the behavior of the encapsulated cells	[100]
MEGEL/PEGDA/alginate ^6^	HAVICs, human aortic valve sinus smooth muscle cells (HASSMCs) ^7^, and human adipose derived mesenchymal stem cells (HADMSCs) ^8^ (2.5 × 10^6^/mL)	extrusion-based bioprinting	variable combinations of photoinitiator type (Irgagure 2959 versus VA086) and concentration, and light intensity (2–136 mW/cm^2^) can be used to optimize cell viability during 3D printing for multiple cell types	[102]
GelMA/HAMA ^9^	human VICs (10 × 10^6^/mL)	extrusion-based bioprinting	a 3D model of calcific aortic valve disease (CAVD) ^10^ was created recapitulating leaflet layer-specific mechanical properties which is useful for studying the valvular mechanobiology and for high-throughput drug screening	[109]

^1^ smooth muscle cells; ^2^ valve interstitial cells; ^3^ methacrylated-hyaluronic acid; ^4^ methacrylated-gelatin; ^5^ human aortic VICs; ^6^ methacrylated gelatin/poly-ethylene glycol diacrylate/alginate; ^7^ human aortic valve sinus smooth muscle cells; ^8^ human adipose derived mesenchymal stem cells; ^9^ gelatin methacrylated/methacrylated HA; ^10^ calcific aortic valve disease.

**Table 3:** Applications of 3D bioprinting for drug screening.

Bioink	Cell (Concentration)	Drug Tested	Significance	Ref.
alginate/GelMA ^1^	human ECs ^2^ (1 × 10^7^/mL) and neonatal rat CMs ^3^ (1 × 10^6^/mL)	doxorubicin (anti-cancer drug)	the doxorubicin dose-concentration response was evaluated in the endothelialized-myocardium-on-a-chip both as beating rate in CMs and as relative expression levels of vWF ^4^ in ECs	[116]
dextran, TPU ^5^, CB ^6^:TPU, Ag:PA ^7^, soft PDMS ^8^, rigid PDMS	iPSCs-CMs ^9^ (220 k/cm^2^)	verapamil (cardiac drug), isoproterenol (cardiac drug)	the engineered microtissues displayed inotropic responses to verapamil and isoproterenol comparable to data obtained from engineered 3D neonatal rat ventricular myocardial tissues and isolated postnatal whole rat hearts	[117]
fibrin-based composite hydrogel (20 mg/mL fibrinogen, 30 mg/mL gelatin, 20 μg/mL aprotinin, 10% glycerol, and 3 mg/mL HA ^10^)	rat CMs (10 × 10^6^/mL)	epinephrine (cardiac drug) and carbachol (cardiac drug)	the bioprinted cardiac tissues physiologically responded to the tested cardiac drugs by modulating the CMs beating frequency; reversible effects of the drugs were observed once these were removed from the bioprinted tissues, thus confirming the effectiveness of these constructs as in vitro 3D tissue models	[118]

^1^ gelatin methacrylated; ^2^ endothelial cells; ^3^ cardiomyocytes; smooth muscle cells; ^4^ von Willebrand factor; ^5^ thermoplastic polyurethane; ^6^ carbon black nanoparticles; ^7^ silver particle-filled, polyamide; ^8^ polydimethylsiloxane; ^9^ cardiomyocytes derived from human induced pluripotent stem cells; ^10^ hyaluronic acid.
